# Streaming Data Fusion for the Internet of Things

**DOI:** 10.3390/s19081955

**Published:** 2019-04-25

**Authors:** Klemen Kenda, Blaž Kažič, Erik Novak, Dunja Mladenić

**Affiliations:** 1 Artificial Intelligence Lab, Jozef Stefan Institute, 1000 Ljubljana, Slovenia; blaz.kazic@ijs.si (B.K.); erik.novak@ijs.si (E.N.); dunja.mladenic@ijs.si (D.M.); 2 Jozef Stefan International Postgraduate School, 1000 Ljubljana, Slovenia

**Keywords:** data fusion, stream mining, machine learning, incremental learning, time-series analysis

## Abstract

To achieve the full analytical potential of the streaming data from the internet of things, the interconnection of various data sources is needed. By definition, those sources are heterogeneous and their integration is not a trivial task. A common approach to exploit streaming sensor data potential is to use machine learning techniques for predictive analytics in a way that is agnostic to the domain knowledge. Such an approach can be easily integrated in various use cases. In this paper, we propose a novel framework for data fusion of a set of heterogeneous data streams. The proposed framework enriches streaming sensor data with the contextual and historical information relevant for describing the underlying processes. The final result of the framework is a feature vector, ready to be used in a machine learning algorithm. The framework has been applied to a cloud and to an edge device. In the latter case, incremental learning capabilities have been demonstrated. The reported results illustrate a significant improvement of data-driven models, applied to sensor streams. Beside higher accuracy of the models the platform offers easy setup and thus fast prototyping capabilities in real-world applications.

## 1. Introduction

The scientific community has been discussing the rising amount of data originating from the internet of things (IoT) for more than a decade. The IoT reached the mass market in early 2014 and its ubiquitous influence and challenges are still permeating the scientific literature. The field of big data processing has improved drastically and a plethora of solutions for various IoT problems have reached their production stage [1].

The volume of the data keeps rising and as the technology is penetrating new markets (i.e., water management), new challenges are put in front of the industry and academia. The need for efficient and accurate analysis of these data is still an issue [2]. Stream processing [3] has been established as a potential answer to the analysis of big data and incremental learning has been rediscovered to answer some of the challenges (like concept drift [4] or learning efficiency [5]). While the field of incremental learning has matured through the last decade and a wide variety of algorithms have been described, tested and implemented in various software libraries, the applications of methodologies from a laboratory to the real world have been scarce. Throughout our work in various applications within the environmental domain, water management, traffic, energy efficiency and smart grid modeling, we have identified the following shortcomings: (i) the most comprehensive software library [6] for stream mining methods is an academic project and therefore requires an additional effort when migrating to production, (ii) modern stream mining frameworks (like Apache Spark, Flink and others) do not implement the state-of-the-art incremental learning methodologies or on-line data fusion strategies; it is also extremely difficult to find an operational implementation of an advanced incremental learning regression, (iii) most of the scientific work on incremental learning has taken place inside the lab, emulating unreal (ideal) conditions, which are rarely encountered in the real world; mostly this remark applies to the data preparation step (including data fusion and generation of machine-learning-ready rich data streams).

Lack of on-line data pre-processing techniques also reduces the possibility of using hybrid approaches, where data pre-processing is done on-line and prediction models are implemented using traditional machine learning (ML) approaches. McKinsey has established that up to 40% of the data value emerging from the IoT is hidden within the synergy effects of different systems [7]. With the exception of the IoT Streaming Data Integration (ISDI) framework [8] which solves time alignment issues of data integration, a generic methodology for generation of feature vectors for machine learning approaches in the IoT scenario does not yet exist and this paper aims to fill this gap. The proposed framework offers a complete streaming methodology for building rich vectors, describing important process characteristics (or features), suitable for traditional or incremental machine learning algorithms. Throughout this document, we will refer to such rich vectors as feature vectors. The proposed methodology is able to merge data from a set of heterogeneous streaming data sources (i.e., from the IoT, weather forecasts and data about human behaviour) in a real-world setting. Our experiments show that this enables machine learning models to yield more accurate and thus more useful results.

In this paper we show use cases related to energy management and traffic, however, the methodology could be useful also in other scenarios such as: algorithmic trading, health care, production line monitoring, intrusion and fraud detection, traffic monitoring, vehicle and wildlife tracking, sports analytics, context-aware promotions and advertising, computer systems and network monitoring, predictive maintenance, geospatial data processing, public transport, public health management, efficient and cost-effective services.

### Motivation and Contributions

For almost two decades the review papers on stream mining [9,10,11,12,13,14] have been identifying the need for proper pre-processing of the data for the needs of stream mining techniques. According to the related work mentioned above, this still remains an open issue. There are systems that enable fast processing or automated data retrieval or single-stream enrichment, however, ensuring semantically correct generation of rich feature vectors from multiple heterogeneous data sources in a streaming scenario has only been partially solved.

Based on our research experience with various applications of stream mining techniques, including prediction of energy consumption in public buildings and smart grids, traffic prediction, prediction of public train energy consumption, spot market price prediction, groundwater levels prediction and others, we have developed a novel approach to be implemented in real-world scenarios. Contributions of our work are as follows:

1. **A formal definition** of heterogeneous data streams fusion. We provide a rigorous mathematical definition of the problem, where we define data streams and operators needed to provide final results—rich feature vectors to facilitate accurate predictive modeling.
2. **A generic streaming data fusion framework** for heterogeneous data streams. To the best of our knowledge, we provide the first generic framework for generation of feature vectors from heterogeneous data streams which supports applications of machine learning techniques in a streaming scenario.
3. **A conceptual architecture** for real-world application of stream mining techniques on heterogeneous multi-sensor data streams. Our experiments extend beyond the laboratory environment and are integrated into real-world scenarios. We propose embedding of the stream fusion framework within big data lambda architecture and its use in the cloud and edge infrastructure.
4. **An improvement of modeling capabilities** of the real-world IoT systems. We demonstrate that the proposed approach improves modeling accuracies in various scenarios. We provide a result-based methodology for evaluation of stream fusion frameworks.

The rest of the paper is structured as follows. Related work is described in Section 2, which is followed by a rigid mathematical problem definition in Section 3. Architecture and methods, used to solve the identified problems, are described in Section 4, integration scenarios are presented in Section 5. Results from real-world use cases are reported and discussed in Section 6. Finally, the paper is concluded in Section 7, where also possible future work directions are presented.

## 2. Related Work

In this section we present a selection of recent use cases, where streaming data fusion has been applied with success. We differentiate between common streaming data fusion methodologies, which integrate domain-knowledge into models, from domain agnostic methodologies, like ours. Streaming data fusion is naturally extended with incremental learning techniques, where we give a basic overview of the state-of-the-art. Finally, we conclude the section with a presentation of academic and production-grade stream processing engines and an overview of comparable streaming data fusion platforms.

**Sensor fusion** is helpful for improving certain functionalities and model accuracy **in various domains**, i.e., in positioning and navigation [15,16,17], in activity recognition [18,19], in system monitoring and fault diagnosis [20,21,22,23,24,25], in transport [26], in health care [27] and in others.

In health care, for example, data fusion is used in IoT-enabled settings such as remote patient monitoring systems. Here, the patient is monitored with different body and environmental sensors, whose signals are processed and used to inform the doctors of the patients condition. Data fusion is used to combine the different signals on three distinct levels: raw level (fusion of raw sensor data), feature level (combining features provided by different methods), and decision level (combining decisions or confidences of medical experts). The fusion is also used for computing context awareness, which is among others used for assigning dynamic roles to doctors. Use of fuzzy logic in context awareness is discussed in [27]. Integration of our framework in a remote patient monitoring system could provide additional improvements (i.e., inclusion of historical data in combination with current values).

Most of the mentioned sensor fusion methodologies expect all the data to be coherent, available immediately and arriving in the correct order. In many localized systems this is the case, however, in the IoT scenarios, the availability of the data contributes to most of the problems. Access control plays an important issue in data management and is a vivid topic in the recent literature [28,29,30]. Our platform builds on top of mechanisms, described in the literature and can take advantage of recent findings, especially those related to streaming platforms. Rare contributions discuss handling of delayed or out-of-sequence measurements [31]. Many of the systems also incorporate significant domain knowledge (model-driven approaches) into the data fusion model (mainly into the Kalman filter’s transition matrix) [32], by which the models lose their generalization potential. Frameworks that have the potential to be applied in various use cases need to be domain knowledge agnostic (purely data-driven), at least with the modeling algorithm [33]. In this sense, any machine learning algorithm acts as a data fusion model since it combines multiple indicators into a single prediction. The idea has been developed further by heterogeneous feature fusion machines [15] that consider mapping multidimensional feature vectors into 1-dimensional output by using classic kernel functions, such as linear, polynomial and Gaussian with different regression methods. With these approaches, the challenge of generating correct and expressive feature vectors to support accurate modeling remains unsolved.

**Big data and stream pre-processing.** In large data sets, where stream mining is the approach of choice, data pre-processing and reduction are becoming critical methodologies for knowledge discovery [14,34]. The authors identify the essential role of such methodologies in efficient machine learning systems. Crucial pre-processing functionalities include concept drift detection and adaptation, missing data imputation, noise treatment, data reduction and efficient and accurate stream discretization algorithms (we refer to these operators as *stream aggregators* in this paper) and imbalanced learning. Automated data analysis is of no use if data pre-processing requires manual intervention [35]. The authors present adaptive pre-processing which benefits the final prediction accuracy on real sensory data. We also use the same evaluation strategy for our methodology, however, our methodology focuses on building rich feature vectors, whereas the paper addresses adaptation to concept drift in the input data stream.

**Stream mining and incremental learning.** Stonebraker and co-authors [36] have identified eight requirements of a stream processing engine (SPE) already in 2005. Among them are a requirement to handle stream imperfections (i.e., delayed or missing data), a requirement to integrate stored and streaming data as well as requirements to keep the data moving and process the data and respond instantaneously. Stream processing engines often base their modeling capabilities on incremental learning methodologies [3,37]. The most popular method for incremental learning is still the Very Fast Decision Tree (VFDT) [38], which has been improved numerous times over the years. An interesting alternative, which is able to learn faster (achieve better accuracy sooner) and converges to batched decision tree form, is the Extremely Fast Decision Tree (EFDT) [39]. Vertical Hoeffding Trees (VHT) are the first distributed streaming algorithm for decision trees and offer significantly improved computation speed in comparison to VFDT and EFDT [40]. A lot of effort has also been dedicated to incremental learning in the deep learning domain [41]. With network architectures that include long short-term memory (LSTM) modules, the problems of heterogeneous data fusion might be at least partially solved already within the learning method. Evaluation of incremental learning techniques is usually achieved with the prequential evaluation approach [42]. Our framework supports such evaluation of incremental learning methods.

**Frameworks for stream processing.** Several architectures and solutions have arisen from the wave of distributed processing engines originating at Hadoop. A couple of generations of Apache domain projects have arisen in the last decade like Apache Spark [43], Apache Samza [44], Apache Flink [45] and Apache Apex [46]. In addition, message distribution systems (like Apache Kafka) have evolved, providing infrastructure for fast stream processing. Some of the systems support the enrichment of data streams with aggregations, some even offer to merge data streams based on the premise, that the most recent data is available immediately in the stream. More complex data fusion strategies are up to the user. Our methodology does provide those missing strategies as well as it implements aggregation operators. All described Apache Software Foundation’s top-level projects take into account distributed processing of data streams, which is not the focus of our research. As we will describe in the following sections, within the IoT, the distributed processing emerges naturally as most often we have to process data from many IoT devices, where each device offers a limited problem, that can be handled within one processing unit.

No efficient production targeted tool mentioned above implements state-of-the-art incremental learning methods. These implementations are still limited to academic community. The most well-known tool for stream mining are MOA (Massive Online Analysis) [6] and its clones in other languages (i.e., streamDM-cpp [47] and scikit-multiflow [48]). While these tools provide implementations of the state-of-the-art stream learning algorithms, they completely ignore the need for on-line data pre-processing and streaming data fusion.

QMiner [49] is a stream processing engine (SPE). It offers operators for aggregating data streams as well as operators for merging and resampling multiple streams. We have built our methodology on top of QMiner infrastructure and extended its functionality to support heterogeneous streaming data fusion.

**Streaming data fusion platforms.** A conceptual platform [50] for the usage of stream mining in the domain of big data is describing a lambda architecture [51] approach. While the authors list all the relevant technologies and mention methods for a summary of streaming data, the platform does not present any details on data fusion implementation.

Real-time probabilistic data fusion for the large-scale IoT applications [26] demonstrates the usage of multi-modal data streams (the IoT data, weather and social media data streams) for efficient prediction of traffic congestions. The method implements a two-level architecture, where the first level analytics derives events from data streams and the second level is essentially a probabilistic complex event processor. They generate the rules with efficient batch processing. They also expose the problem of using a common time scale for heterogeneous data sources but exclude the possibility of delayed measurements. A similar approach is described in [52], where the authors formalize the position of machine learning/analytics within the *hut* architecture, where it is used to support event processing by providing rules through batch analytics. Both approaches, however, perform this operation in the batch processing part. On the contrary, our approach includes machine learning methodologies in the streaming part of the architecture and introduces incremental learning approach, which can work without the support of the batch processing part.

Autonomous discovery of high-level knowledge from ubiquitous data streams [16] is one of the rare works that does not focus only on combining specific information with well-defined meaning, but rather tries to provide a general framework, agnostic to a specific problem. The authors use data aggregation over time to summarize detailed data streams (and their derivatives) and provide fixed feature vectors based on *n* uniformly sampled data streams. In addition, we provide a general framework that is able to ingest multiple heterogeneous (non-uniformly sampled) data streams and is able to provide user-defined feature vectors based on current as well as historical aggregates over the data and their derivatives.

Multiple streams data fusion is presented in [53]. The methodology exploits multiple sensors measuring the same property to predict anomalies and does not attack the issue of heterogeneous streams data fusion. The IoT streaming data integration (ISDI) paradigm is introduced in [8] and the proposed ISDI framework solves real-time data integration using the generic window-based algorithm. The work addresses the crucial timing alignment issue in the IoT setting. While both, ISDI and our framework, solve similar issues, our proposal includes a solution that works in a truly streaming manner (using a single-pass over data records), includes integration of historical values and provides a more direct interface for generation of stream aggregates.

Data fusion is one of the central research topics within the IoT, however, rare domain agnostic platforms for the fusion of the heterogeneous streaming data sources which support machine learning techniques have been presented in the scientific literature so far. Related contributions have, however, increased noticeably over the last couple of years.

## 3. Problem Definition

One of the exploitation scenarios for the vast IoT data is to take advantage of its predictive potential through machine learning methods. For example, based on historical data from a smart grid we can build a model that is capable of predicting energy consumption profiles for the next day, which will help better planning of the energy distribution and thus provide cheaper energy for the end user. In order to create the best possible predictions we need to be able to not only work with the current power consumption values, but also with historical data, different stream aggregates and derivatives and, what is even more important, we need to be able to expand the data streams with relevant contextual information, such as weather data, human behaviour data and weather forecasts. Moreover, an on-line algorithm for creating rich feature vectors for machine learning methodologies is needed in order to provide new feature vectors as soon as possible and to support incremental learning scenarios. Our framework builds such feature vectors and exposes them to machine learning methods. Incremental learning methods are well tailored to the needs of the IoT since the models are computationally cheaper and since they usually capture concept drift (change of statistical properties of the target variables), which often appears in the IoT scenarios.

The two dominant reasons why this kind of data fusion task is not trivial are the heterogeneity of the IoT data and its time incoherence.

According to [54], heterogeneity is an intrinsic property of big data. Many definitions of heterogeneous data can be found in the literature and there is no common agreement on the definition among various authors. Among the properties that illustrate the issue are: Multi-modality of the data (even considering a mixture of continuous and categorical features and structured and unstructured data) and the technical aspects (i.e., format of the data), rate of independence, concept drift and dynamics of change, and privacy. In this work we consider data coming from different sources and focus on heterogeneity based on the discrepancies in the time component [55,56], which is, in our opinion, the most important in the IoT data streams. It is manifested through the following properties: (i) sampling frequency, (ii) time delay, and  (iii) data availability. Sampling frequency differs from sensor to sensor. Some sensors implement constant sampling frequency. Different sensors within a setup could implement constant, but different sampling frequencies. Many sensors implement approximately constant sampling frequency. he readings happen approximately in the prescribed interval, but due to different effects, the reading might be slightly early or late. Some sensors might use arbitrary sampling frequencies (i.e., they might only report an event). Time delay is introduced with transmission latencies, legacy systems and privacy/access issues. Measurements might be late from a few milliseconds up to one day (i.e., when data is transmitted from a legacy system via FTP connection). Delay is closely related to data availability.

A data stream is a sequence of values with a corresponding timestamp (in IoT a timestamp denotes the time when the measurement was taken). We define a coherent time series to be such a sequence, where each subsequent measurement in a series has been taken later than the previous. The most obvious data source which breaks time coherence is the weather forecast data stream. The forecasting models update their predictions regularly, usually every hour. With every update, older forecasts are updated with more accurate values, based on recent data.

Based on the issues arising from heterogeneity of the data we define a harmonic set of data streams. A harmonic set of data streams consists of a set of data streams, where each stream has a matching sampling frequency (and phase), at least one matching timestamp, and all the data that is needed for the successful generation of a feature vector.

The following subsections (Section 3.1, Section 3.2, Section 3.3, Section 3.4, Section 3.5, Section 3.6 and Section 3.7) present different types of data sources and their main characteristics as well as a thorough mathematical formulation of the basic concepts and approaches used in our methodology.

### 3.1. Types of Data Sources

Modeling in the IoT scenarios is based on the three different types of data sources. (1) Sensor data. Sensor data most often originates from IoT devices but can be obtained also by crawling a particular web resource. The data is usually obtained close to real-time. However, different lags can be introduced due to various reasons. The data fusion system should be able to handle these time-related inconsistencies and build correct feature vectors, based on the most recent available data. (2) Weather forecasts. Weather forecasts are available for the future (usually we need them up until the time of our prediction horizon). The forecasts represent an incoherent data stream (as the values are updating with time), which needs to be handled appropriately in the stream fusion system. (3) Static data. This is the data that by definition does not change in time. Values are known for the indefinite future. The data includes attributes like: day of the week, day in the year, hour of the day, holiday, day before the holiday, working hour, etc. For simplicity reasons, we handle static data as a stream.

**Figure 1:**
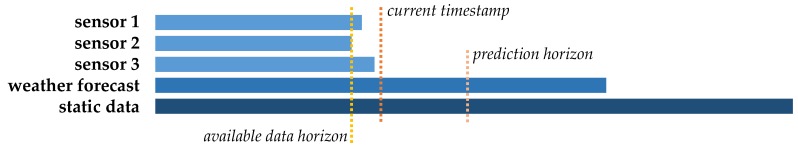
Data availability of different types of data sources. Sensor data is delivered in (almost) real-time. However, some legacy systems might introduce longer lags. Weather forecasts are available for a particular time in the future, while static data (i.e., date/time features and human behaviour data) are usually always available.

Availability of the three different types of data differs as depicted in Figure 1. Depending on the delivery mechanisms, sensor data arrives to the stream processing components with different lags. Figure 1 also introduces the available data horizon which is the latest timestamp for which all sensor data streams are available. It represents the latest timestamp for which feature vector generation can be triggered. Feature vectors are built for calculating predictions at the prediction horizon. Static and weather forecast data are therefore usually considered for that timestamp within feature vectors.

### 3.2. Data Streams

In most literature a data stream is represented by a sequence of values $x_{1},x_{2},\ldots ,x_{n}$ where $x_{i}\in R$ is an observation for all i∈{1,2,…,n}. We recognize that this notation has a drawback: it does not contain any information about when a particular value has been provided. Time is an important factor in deciding when to start a particular process on the data stream. To that end, we present a new definition of a data stream that includes time.

A **data stream** $O_{x}^{n}$ is an ordered set of value-time observation pairs provided by a sensor or some other source of data and is described as

$$O_{x}^{n}=\{\left(x_{1},t_{x}^{\left(1\right)}\right),\left(x_{2},t_{x}^{\left(2\right)}\right),\ldots ,\left(x_{n},t_{x}^{\left(n\right)}\right)\},$$

where $t_{x}^{\left(k\right)}$ is the time of observation $x_{k}$ and $t_{x}^{\left(i\right)}\leq t_{x}^{\left(j\right)}$ for all i≤j. A **data stream window** $O_{x}^{i,j}$ is a subset which contains observations between the *i*-th and *j*-th entries of a data stream $O_{x}^{n}$ and is described as

$$O_{x}^{i,j}=\{\left(x_{i},t_{x}^{\left(i\right)}\right),\left(x_{i+1},t_{x}^{\left(i+1\right)}\right),\ldots ,\left(x_{j},t_{x}^{\left(j\right)}\right)\}.$$

In addition, we will write $O_{x}^{1,n}=O_{x}^{n}$.

**Remark** **1.** *A data stream can also be defined as an ordered set of vector-time observation pairs, e.g., by replacing the values with vectors in the definition above. By doing so we would allow an observation to contain multiple values and thus generalize the data stream. For the sake of simplicity, we will use the value-time data stream definition in the rest of the document and will reference this remark when required.*

### 3.3. Static Data Stream

Data streams are dynamic in nature; they are created by retrieving signals from sensors which are then transformed and added to the stream. In some cases, we know what data will come at a particular timestamp. One such case is the day-of-the-week data stream where for a given timestamp we know which day of the week it corresponds to. This type of data streams is defined as static data streams $S_{w}^{n}$ and is described as

$$S_{w}^{n}=\{\left(w_{1},t_{w}^{\left(1\right)}\right),\ldots ,\left(w_{n},t_{w}^{\left(n\right)}\right)\}.$$

Returning to the day-of-the-week static data stream, it contains values $w_{i}\in \left[1,2,\ldots ,7\right]$ where the number corresponds to a particular day of the week associated with its timestamp (1 corresponding to Monday, 2 to Tuesday etc.). Another example is the *weekend static data stream* which contains information weather a timestamp is in a weekend interval. Its values are $w_{i}=1$ if the timestamp $t_{w}^{\left(i\right)}$ is inside a weekend interval and $w_{i}=0$ otherwise. Similarly, a *holiday static data stream* is a static data stream which contains information if a timestamp falls in a holiday. Notice that some data streams depend on the context (e.g., culture, country).

### 3.4. Data Stream Aggregate

When we process data streams we might want to group observations together to form a new value that summarizes the data stream. To do this, we require a data stream **aggregate** function which is able to combine the data stream observations and returns the summarized (aggregated) value. A data stream aggregate can also be applied on a data stream window $O_{x}^{i,j}$. Generally, a data stream aggregate function is defined as

$$\mathrm{aggr}(O_{x}^{n})=X,$$

where *X* is the aggregated value of the provided observations. The most common data stream aggregate functions are (a comprehensive list is available in [57,58]):

- **Count.** Counts the number of observations in a data stream: X=n,
- **Maximum.** Returns the maximum value in a data stream: $X=\max\{w_{1},\ldots ,w_{n}\}$,
- **Minimum.** Returns the minimum value in a data stream: $X=\min\{w_{1},\ldots ,w_{n}\}$,
- **Sum.** Sums up all values in a data stream: $X=\sum_{i=1}^{n}w_{i}$.

A more complex example of a data stream aggregate is the **moving average** (MA). This aggregate is used to smooth out short-term fluctuations and highlight longer-term trends. It calculates the average of the observations within a data stream window $O_{x}^{i,j}$ and is defined as

$$\mathrm{MA}\left(O_{x}^{i,j}\right)=\frac{1}{j-i+1}\sum_{k=i}^{j}x_{k}.$$

When the data stream window moves the new MA can be calculated by using the previous MA value:

$$\mathrm{MA}\left(O_{x}^{i+1,j+1}\right)=\frac{\left(j-i+1\right)\cdot \mathrm{MA}\left(O_{x}^{i,j}\right)-w_{i}+w_{j+1}}{j-i+1}$$

The second more complex aggregate function is the **exponential moving average** (EMA). It is similar to MA only that it incorporates a decaying factor; giving the more recent observations greater importance. This aggregate inputs the data stream $O_{x}^{n}$ and is calculated with the following recursive function:

$$\mathrm{EMA}\left(O_{x}^{n}\right)=\left\{\begin{array}{cc} x_{n}, & \mathrm{for}n=1,\\ \alpha \left(n\right)\cdot x_{n}+\left(1-\alpha (n)\right)\cdot \mathrm{EMA}\left(O_{x}^{n-1}\right), & \mathrm{for}n\neq 1, \end{array}\right.$$

where $\alpha \left(n\right)=\frac{\Delta t(n)}{T}$, $\Delta t\left(n\right)=t_{x}^{\left(n\right)}-t_{x}^{\left(n-1\right)}$ represents the rate of the decay and *T* is the user defined split time constant.

Once we decide on the aggregate functions to use in processing, we can create an aggregated data stream. An **aggregated data stream** $O_{x,\mathrm{aggr}}^{n}$ is a data stream containing the sequence of aggregated values of $O_{x}^{n}$ by using the aggregate function “aggr”.

$$O_{x,\mathrm{aggr}}^{n}=\left\{(\mathrm{aggr}\left(O_{x}^{1}\right),t_{x}^{\left(1\right)}),\ldots ,(\mathrm{aggr}\left(O_{x}^{n}\right),t_{x}^{\left(n\right)})\right\}.$$

We will now look at two aggregated data stream examples:

**Moving average data stream.** This aggregated data stream is created by using the MA aggregate and is described as

$$O_{x,\mathrm{MA}}^{n}=\left\{(\mathrm{MA}\left(O_{x}^{1,k+1}\right),t_{x}^{\left(1\right)}),\ldots ,(\mathrm{MA}\left(O_{x}^{n-k,n}\right),t_{x}^{\left(n\right)})\right\},$$

where k<n is the user defined data stream window size.

**Exponential moving average data stream.** This aggregated data stream is created by using the EMA aggregate and is described as

$$O_{x,\mathrm{EMA}}^{n}=\left\{(\mathrm{EMA}\left(O_{x}^{1}\right),t_{x}^{\left(1\right)}),\ldots ,(\mathrm{EMA}\left(O_{x}^{n}\right),t_{x}^{\left(n\right)})\right\}.$$

More complex stream aggregates can require interpolation over the time-series and calculation of values such as: number of extremes in a particular data stream window, highest *n*-th derivative in the data stream window, duration of the largest maximum, etc. Such derivatives are, for example, useful for modeling of crop types in earth observation scenarios.

### 3.5. Data Stream Resampler

One of the properties of data streams is that observations might not come at a constant rate. This can cause problems when multiple data streams need to be synchronized. To handle this issue we define a **sampling function** which takes a data stream $O_{x}^{n}$ and a timestamp *T* as an input and returns a sample value. The function can be described as

$$\mathrm{sampler}\left(O_{x}^{n},T\right)=X_{i},$$

where $X_{i}$ is the sampled value generated from the input parameters. The sample value can be generated using different functions:

- **Last value.** This function returns the last observation that appeared before the provided time: $x_{k}$, where $t_{x}^{\left(i\right)}<T$ for all i≤k and $T\leq t_{x}^{\left(j\right)}$ for k<j.
- **First value.** This function returns the first observation that appears after the provided time: $x_{k}$, where $t_{x}^{\left(i\right)}<T$ for all i<k and $T\leq t_{x}^{\left(j\right)}$ for k≤j.
- **Linear interpolation.** This function returns the sample value by using a linear interpolation between observations around the provided time, e.g., 
  $$\mathrm{lin}\left(O_{x}^{n},T\right)=\frac{x_{k}-x_{k-1}}{t_{x}^{\left(k\right)}-t_{x}^{\left(k-1\right)}}\left(T-t_{x}^{\left(k\right)}\right)+x_{k},$$
  where $t_{x}^{\left(k-1\right)}\leq T\leq t_{x}^{\left(k\right)}$.

Once we decide on the sampling function *f* and a constant time period *T* we can create a data stream of sampled data. This type of data streams is defined as a **resampled data stream** containing the sampled values of a provided data stream $O_{x}^{n}$ and is described as

$$O_{x,f}^{m}=\left\{\left(X_{1},T_{X}^{\left(1\right)}\right),\ldots ,\left(X_{m},T_{X}^{\left(m\right)}\right)\right\},$$

where $X_{i}=f(O_{x}^{n},T_{X}^{\left(i\right)})$ is the sampled value returned by the sampling function *f* performed at time $T_{X}^{\left(i\right)}$. In addition, the difference of consecutive time values is equal to the constant time period *T*, i.e.,

$$T=T_{X}^{\left(i+1\right)}-T_{X}^{\left(i\right)},$$

for all i∈{1,…,m−1}.

### 3.6. Data Stream Merger

Sometimes we require to merge two or multiple data streams into a single data stream. We define a merger function that is able to do just that. Suppose we have two data streams $O_{x}^{n}$ and $O_{y}^{m}$ where $t_{x}^{\left(i\right)}=t_{y}^{\left(i\right)}$ for all i≤min{n,m}. A **merger function** takes $O_{x}^{n}$ and $O_{y}^{m}$ as an input and returns the merged data stream, i.e.,

$$\mathrm{merger}\left(O_{x}^{n},O_{y}^{m}\right)=\{\left(x_{1},y_{1},t^{\left(1\right)}\right),\ldots ,\left(x_{k},y_{k},t^{\left(k\right)}\right)\},$$

where k=min{n,m} and $t^{\left(i\right)}=t_{x}^{\left(i\right)}=t_{y}^{\left(i\right)}$ for all i∈{1,…,k}. If we adopt the view provided in Remark 1, the output of the merger function is a data stream. Indeed, if we write the values $x_{i}$, $y_{i}$ as entries of a vector then the output will follow the generalized definition of a data stream.

### 3.7. Forecast Data Stream

Forecast data streams provide forecasted values for the future. The difference between the time of the forecast and the time of the forecast generation is called a prediction horizon. A simple forecasting model provides forecasts for a constant prediction horizon and can be described simply by $O_{p}^{n}$, where $p_{i}$ is the forecasted value and $t_{p}^{\left(i\right)}$ refers to a timestamp in the future. More complex forecasting streams (i.e., weather forecasts) provide predictions for multiple time horizons at the same time. For example, every hour new weather forecasts are being generated for the next 48-hour interval. This implies that a forecast for a particular hour in a day gets updated 48-times during this process. At forecast generation a new data stream window $O_{p}^{i,j}$ is generated, thus the forecast data stream is defined as

(1) $$F_{x}^{n}=\left\{\left(O_{p}^{1,k},t_{x}^{\left(1\right)}\right),\left(O_{p}^{2,k+1},t_{x}^{\left(2\right)}\right),\ldots ,\left(O_{p}^{n,k+n-1},t_{x}^{\left(n\right)}\right)\right\},$$

where *k* is the number of time horizons and $O_{p}^{i,k+i-1}$ is the data stream window generated at time $t_{x}^{\left(i\right)}$. In the case of k=1, the forecast data stream contains a simple forecasting model, which generates only one prediction of a constant prediction horizon.

### 3.8. Feature Vector

In machine learning, a *feature vector* is a vector that contains data important for description and modeling of a particular system. Together with a label of a particular data instance, it is used for training of a machine learning model. If the label is unknown, the vector can be used to derive predictions (or other results) from a trained model. An element in the vector is called a feature (in some literature it is referred to as an attribute). A feature vector which includes contextually rich information, derived from a set of data streams, will allow a machine learning model to achieve the best possible results in modeling particular phenomena. Such a vector $\phi_{\mathrm{full}}$ should be the final result of a streaming data fusion framework.

A feature vector $\phi_{\mathrm{full}}$ is a representation of a set of observation, static data and forecast data streams $O_{x}^{n}$, $S_{y}^{n}$ and $F_{z}^{n}$, where $x\in \{x_{1},\ldots ,x_{i}\}$, $y\in \{y_{1},\ldots ,y_{j}\}$ and $z\in \{z_{1},\ldots ,z_{k}\}$. We will focus on feature vectors of the following form:

$$\phi_{\mathrm{full}}=\left[\begin{array}{c} F^{\left(1\right)}\\ F^{\left(2\right)}\\ \vdots \\ F^{\left(q\right)} \end{array}\right],$$

where *q* is the size of the feature vector and each feature $F^{\left(r\right)}$ is defined with:

$$F^{\left(r\right)}=\left\{\begin{array}{cc} x_{i}^{\left(j\right)}, & a\left(\mathrm{resampled}\right)\mathrm{measurement}\mathrm{extracted}\mathrm{from}O_{x_{i}}^{n}\\ y_{i}^{\left(j\right)}, & a\left(\mathrm{resampled}\right)\mathrm{forecast}\mathrm{extracted}\mathrm{from}F_{y_{i}}^{n}\\ z_{i}^{\left(j\right)}, & a\left(\mathrm{resampled}\right)\mathrm{forecast}\mathrm{extracted}\mathrm{from}S_{z_{i}}^{n}\\ X_{i}^{\left(j\right)}, & a\left(\mathrm{resampled}\right)\mathrm{stream}\mathrm{aggregate}\mathrm{extracted}\mathrm{from}O_{x_{i}}^{n}\\ Y_{i}^{\left(j\right)}, & a\left(\mathrm{resampled}\right)\mathrm{stream}\mathrm{aggregate}\mathrm{extracted}\mathrm{from}F_{y_{i}}^{n}\\ Z_{i}^{\left(j\right)}, & a\left(\mathrm{resampled}\right)\mathrm{stream}\mathrm{aggregate}\mathrm{extracted}\mathrm{from}S_{z_{i}}^{n} \end{array}\right.$$

Note that the index j∈{1,…,n} can refer to current or historical values of the measurements or the aggregates. Additionally, each feature vector reflects the state of the observed system at resampled timestamp $T_{x}^{\left(n\right)}$.

## 4. Architecture and Methods

### 4.1. Architecture

**Figure 2:**
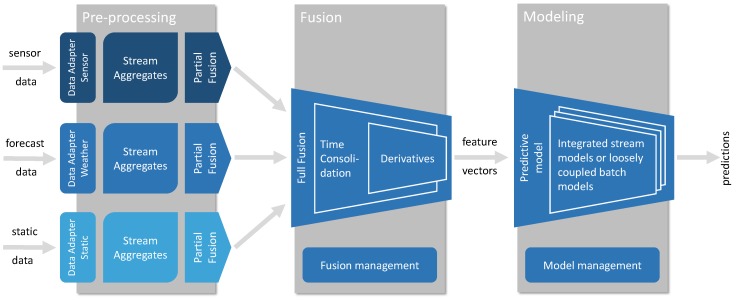
Data fusion framework architecture with added modeling component. The framework consists of three main components: pre-processing, fusion and modeling. Pre-processing is dedicated to the independent transformation of particular data streams, fusion merges them together into full feature vectors, whereas modeling provides predictions from either generated batch models or from incremental learning models.

The architecture of the proposed framework (http://github.com/klemenkenda/iot-fusion/) is depicted in Figure 2. The framework is designed to be easily integrated into a speed layer of a standard big data processing lambda architecture [51]. The proposed framework consists of three main building blocks: pre-processing, fusion and modeling. Pre-processing includes data adapters for various data streams. Data streams are enriched with stream aggregates, resampled to a common timestamp (see Algorithm 1) and partial feature vectors are extracted in the partial fusion component (see Algorithm 2). The data fusion block accepts partial feature vectors from a set of data sources and merges them together into a full feature vector (see Algorithm 3). In this component, potentially different timestamps are compensated and additional derivatives are calculated from partial feature vectors (i.e., the difference between current and yesterday’s daily average electricity consumption).

The modeling component is also included in the framework since data-driven modeling methods perform a kind of data fusion by mapping feature vectors into predictions. Two different modeling components have been implemented: (a) based on a rich ecosystem of batch learning techniques and (b) based on a sparse implementations of stream learning techniques. The latter component is useful in the edge scenarios, where computational efficiency is paramount.

The whole process is controlled via a single configuration structure, which defines the input data sources, data enrichment (a set of stream aggregates attached to a particular data stream), resampling time, the particular elements of a full feature vector and even meta-data relevant for predictive analytics (prediction horizon, selected modeling method and corresponding parameters).

### 4.2. Complex Forecast Transformation into a Coherent Data Stream

Forecasts usually represent important contextual information regarding the process we are trying to model and can drastically improve the accuracy of our models. We have defined the forecast data stream $F_{x}^{n}$ in Equation (1). An attentive reader might observe that such a data stream is breaking the basic rules of a coherent data stream [36]. A forecasted value $p_{j}$ at time $t_{p}^{\left(j\right)}$ gets updated with each new received data stream window $O_{p}^{i,k+i-1}$, as long as j∈{i,i+1,…,i+k−1}. For such streams it is impossible to use the majority of the stream mining algorithms, including stream aggregators.

We propose decomposing the complex stream from Equation (1) into a set of *k* streams, where each stream represents forecast for a constant prediction horizon *h* and can, therefore, be simply denoted as $O_{p_{h}}^{n}$. For example, a weather forecast stream which contains 48-hourly forecasts in each update would decompose into 48 separate coherent data streams. With this transformation complex forecast streams can be simply integrated into our framework.

### 4.3. Data Flow

**Figure 3:**
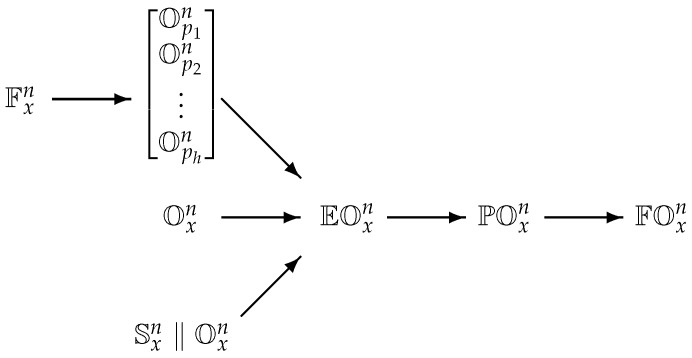
Hierarchy of data streams in the stream fusion framework. Heterogeneous data streams are consolidated and merged with every step of the stream fusion (depicted from left to right). Raw data streams ($F_{x}^{n},O_{x}^{n}$ and $S_{x}^{n}$) are transformed, enriched and resampled into coherent data streams ${\mathrm{EO}}_{x}^{n}$ and then fused through partial feature vector streams ${\mathrm{PO}}_{x}^{n}$ into a final full feature vector data stream ${\mathrm{FO}}_{x}^{n}$.

Data flows within our system reflect the proposed architecture and the particularities of different stream types. On the farther left side of Figure 3 is the forecast complex stream $F_{x}^{n}$. As this stream breaks the definition of a coherent time series it is conformed to a set of simple streams within the data adapter as described in the previous subsection. The second column of Figure 3 represent the initial phase of data fusion. The depicted data streams share the same form but do not have a common time denominator. Afterwards, all data streams are enriched with stream aggregates. Common time denominator is established in streams ${\mathrm{EO}}_{x}^{n}$ with resampling the data streams to to the master time interval *T*. Data fusion is performed in two steps. Firstly, the enriched data streams are transformed into partial feature vector streams ${\mathrm{PO}}_{x}^{n}$ by adding additional historical values or derivatives to the data stream. Finally, these feature vector streams are merged into final feature vector data stream ${\mathrm{FO}}_{x}^{n}$.

### 4.4. Enrichment of a Data Stream

Enrichment of a data stream is a process which takes a data stream $O_{x}^{n}$ as an input and adds a set of additional stream aggregates $O_{x,\mathrm{aggr}}^{n}$ to it. Our enrichment procedure is described in Algorithm 1. Alongside the data stream $O_{x}^{n}$ the algorithm also requires information about the configuration of the stream aggregates (config). Based on the config the algorithm initiates a set of stream aggregates and attaches them to the data stream, which enables stream aggregate operators to update their values with every new element in stream $O_{x}^{n}$. Additionally, we initiate a resampler operator in this step. The task of the resampler operator is to put the data stream $O_{x}^{n}$ to a common time $T_{x}^{\left(i\right)}$, which is shared among all the data streams in the stream fusion process. Enrichment algorithm listens to the data stream and triggers an action after every measurement is received. Firstly, all the attached stream aggregates are updated with the new value. Next, an enriched vector $b_{n}$ consisting of the original measurement, all of the stream aggregate values and the measurement timestamp is created. Finally, the enriched vector $b_{n}$ is inserted into the resampler and if a new resampled vector $b_{n}^{\prime }$ is available it is pushed into the resulting enriched resampled data stream ${\mathrm{EO}}_{x}^{n}$. Note: the data stream $O_{x}^{n}$ only utilizes last values, which means that historical values are not stored by the algorithm. Historical values needed for the calculation of window based data stream aggregates are stored in buffers within the aggregate mechanism [49]. The data stream ${\mathrm{EO}}_{x}^{n}$ is stored as a buffer ${\mathrm{EO}}_{x}^{i,j}$, where the window’s right limit *j* is increasing with each new measurement, and left limit *i* is increased after the cleanup of the obsolete data in Algorithm 3. After the initial phase, when old measurements are being collected in order to satisfy the needs of a feature vector generation, the size of the ${\mathrm{EO}}_{x}^{i,j}$ buffer remains constant.

**Algorithm 1:** Enrichment of a data stream with stream aggregates.

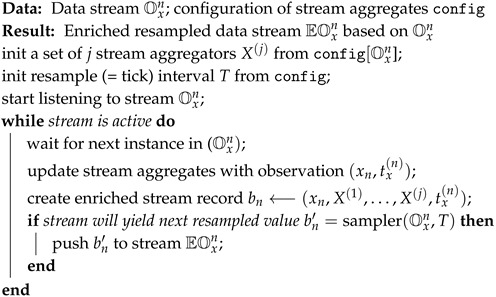



The new stream ${\mathrm{EO}}_{x}^{n}$ satisfies all the properties of a data stream from Section 3 and can be further used in any stream mining algorithms within the pipeline. The new stream contains new enriched data as well as conformed timestamp.

### 4.5. Data Fusion Algorithms

Streaming data fusion consists of two separate algorithms. The partial fusion algorithm (see Algorithm 2) and full fusion algorithm (see Algorithm 3). The partial fusion algorithm transforms an enriched data stream ${\mathrm{EO}}_{x}^{n}$ into a partial feature vector based on the values from this data stream. The full fusion algorithm merges all partial feature vectors together and forms the final full feature vector, which is ready to be used for learning or prediction in any machine/stream learning method.

The purpose of the partial fusion algorithm is to add additional historical and derived features (out of a single data stream) by using a data stream window ${\mathrm{EO}}_{x}^{i,j}$ of recent values. Additionally, the algorithm takes current feature generation time $T_{c}$ and configuration of features (A) as input data. Each feature is identified by an elementPosition (column) in an item of ${\mathrm{EO}}_{x}^{i,j}$ as well as by its relativeOffset from feature generation time in the stream (row). For example, a relevant feature for most of the models, which predict human behaviour (i.e., energy consumption), is the value of the phenomena we are trying to predict from a day before. For instance, if our resampling time *T* is equal to 1 h, the relative offset would be −24. Partial fusion is an algorithm that is called by Algorithm 3. The algorithm initiates an empty partial feature vector *p*. Then it transverses all the features from a set of partial feature vector features A and inserts the appropriate values (according to position and offset) into the partial feature vector. Algorithm 2 can easily be extended with additional feature generators (i.e., time differences or averages over a set of features), which we have demonstrated in the real-world use cases (see Section 6). If the data in the current enriched resampled stream window ${\mathrm{EO}}_{x}^{i,j}$ is inadequate (i.e., some historical data is missing), the algorithm throws an exception. Final partial feature vector $p\in {\mathrm{PO}}_{x}^{n}$ is returned to the full fusion algorithm.

**Algorithm 2:** Partial fusion algorithm in pre-processing step.

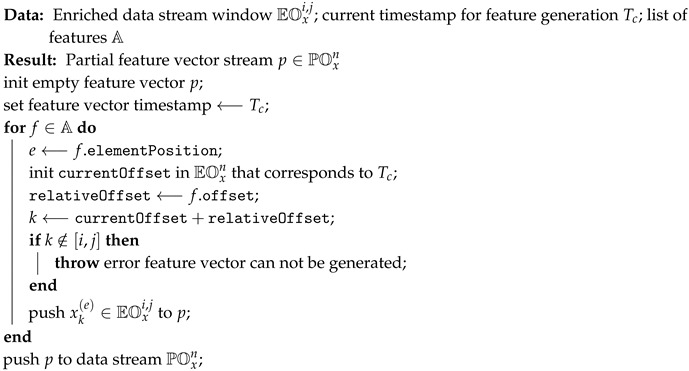



**Algorithm 3:** Full data fusion algorithm.

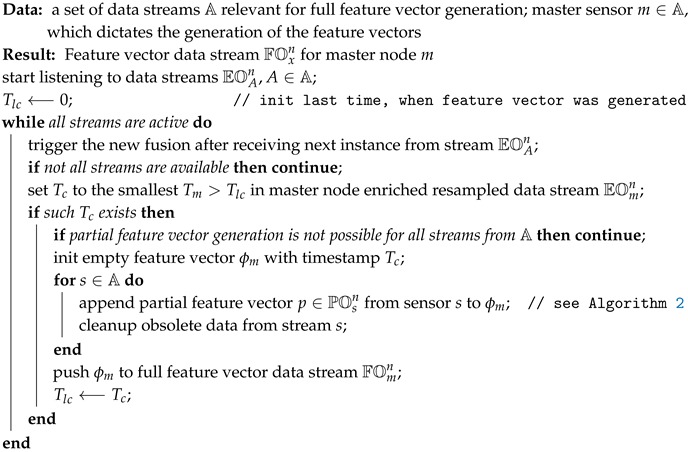



The full data fusion algorithm collects all the partial feature vectors from set A and merges them together in a single full feature vector, which is suitable for usage in various stream/machine learning algorithms. The system requires initialization from a configuration of streams A, relevant for feature vector generation, and information on the master sensor m∈A, which dictates the generation of feature vectors. The result of the algorithm is a full feature vector $\phi_{m}\in {\mathrm{FO}}_{m}^{n}$. The algorithm initiates with the smallest possible time $T_{lc}=0$, which indicates the last time of a feature vector generation. Upon each new record from any of the data streams ${\mathrm{EO}}_{A}^{n}$ the system checks if there is data already available from all streams A∈A. The system tries to identify the smallest possible next feature generation time from master sensor *m*. This is the smallest timestamp for which a full feature vector has not yet been successfully generated. If such a timestamp $T_{c}$ exists, then the algorithm checks whether there is enough data (historical and for the future) within the ${\mathrm{EO}}_{A}^{n}$ buffers for successful generation of partial feature vectors. Then the algorithm instantiates an empty feature vector *p* for timestamp $T_{c}$ and fills it with partial feature vectors from all the relevant sensors. In order to keep the memory usage as low as possible, the algorithm checks for any obsolete data records in buffers ${\mathrm{EO}}_{A}^{i,j}$ and removes them. Finally, the final full feature vector $\phi_{m}$ for the master node *m* is pushed to the appropriate stream ${\mathrm{FO}}_{m}^{n}$, where it is made available for any interested consumers, i.e., stream or batch machine learning algorithms.

The methodology implements a fully streaming data fusion algorithm. The requirement for historical data is satisfied with the usage of smallest possible buffers (i.e., internal buffers of stream aggregates based on sliding window or resampled enriched data stream window ${\mathrm{EO}}_{x}^{i,j}$). The methodology is generic and can be initiated for any stream modeling scenario with a configuration structure, which includes (a) a set of fusion meta-data like fusion id, tick time interval *T* and others, (b) a set of stream aggregates for a particular sensor *s*, where each stream can implement a set of *tick-based* (last value only) or *sliding window-based* aggregates, and (c) the definition of a feature vector, which consists of partial feature vectors for a particular sensor. Each partial feature vector can include an arbitrary set of features, which include current or historical sensor values, current and historical aggregated values over different sliding windows and even derivatives of current and historical values.

## 5. Integration

Our methodology is suitable to be included in lambda and similar big data architectures [51,52]. These architectures define the role of 2-fold data processing layers: batch and speed. Speed layer is dedicated to the processing of data streams. Quite often the speed layer is reduced to event processing [52], however, we propose to use incremental learning techniques independently in the speed layer (at least for edge processing applications) or to transfer the models from batch layer (where they are learned) to the speed layer (where they are used to provide predictions on an almost real-time data stream).

In the following subsections, we present two different integration scenarios: (1) integration in the cloud infrastructure and (2) integration in the edge/fog infrastructure. Additionally, due to the relatively low computational cost of streaming data fusion and modeling components, the system would be ideal for deployment in dew architecture (low-end servers, deployed near IoT devices), as proposed in [59].

### 5.1. Integration: Cloud Infrastructure

**Figure 4:**
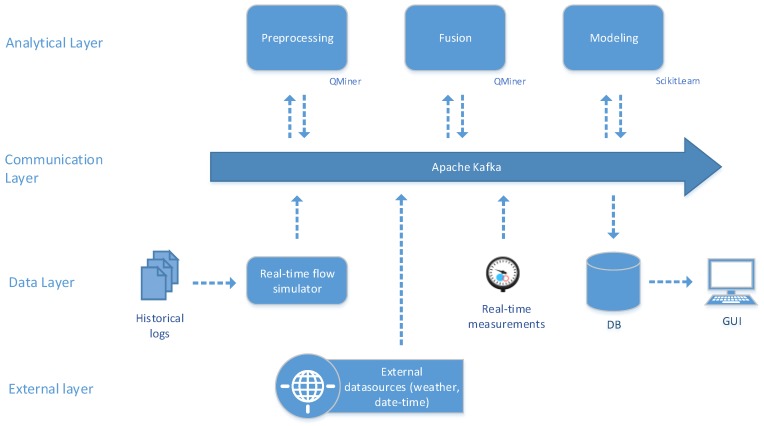
Integration in the cloud infrastructure is based on the message queue in the Communication Layer (in our applications this was Apache Kafka). Each component in the architecture is loosely coupled to the system and receives/sends data to the system via the message queue using a predefined data format. The system can receive simulated or real-time data (from sensors and from external data sources). Results are stored in the monitoring database (DB) and finally shown to the user (GUI).

Integration in the cloud represents the usual implementation of our framework. We have used this setup in smart grid power, power station demand, groundwater level, public buildings power demand and other real-world scenarios. As depicted in Figure 4, such an integration consists of 4 different layers: Analytical Layer, Communication Layer, Data Layer and External Layer. The central component of the system is the message queue system within the Communication Layer. In our implementations, we have used Apache Kafka. Exposed through a single entry point Apache Kafka can distribute extreme amounts of data via elastic, scalable and fault-tolerant infrastructure. As each of the processing components (Preprocessing, Fusion and Modeling) only needs to access the message queue, they can be distributed over the computational infrastructure and thus ensure scalability even in the processing end.

Data is provided in the Data and External Layers. The External Layer includes external data sources, such as weather data, weather predictions and static data (data-time features, human behaviour data, etc.) while the Data Layer includes the essential IoT data infrastructure. Before real-world systems are deployed to the production, extensive testing is needed. This is ensured by real-time flow simulator component, which is able to simulate conditions in the real-time (or faster) based on historical log data files. The platform provides three types of results: (i) pre-processed feature vectors from the pre-processing component, (ii) final feature vectors from the fusion component and (iii) predictions from the modeling component. All the intermediate, as well as final results, are stored in the monitoring database (DB) for further analysis and visualization in the Graphical User Interface (GUI).

We have implemented our stream fusion system in QMiner [49] stream processing engine, which enables fast prototyping as well as production grade framework deployment and already a rich ecosystem of implemented stream aggregate operators.

### 5.2. Integration: Edge/Fog Infrastructure

**Figure 5:**
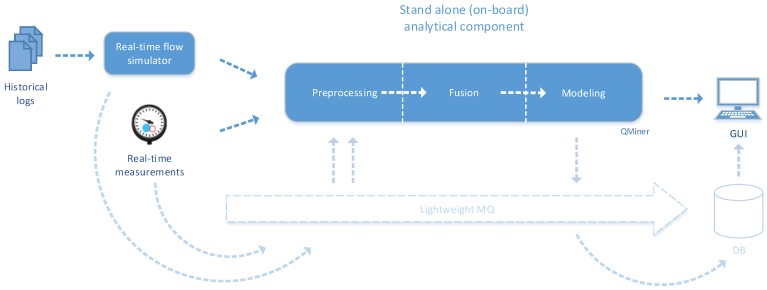
Data fusion framework integration in the edge/fog scenario. Message queue system can be completely omitted in this scenario and all communication is achieved via HTTP API or WebSockets (on the GUI part). Components are tightly coupled to ensure faster data transfer between components. Modeling is included in the analytical component and implements lightweight incremental learning algorithms.

Stream mining systems are suitable for the implementation in the edge/fog infrastructure due to their low computational demand. We have used this setup in energy demand prediction on a public train. As depicted in Figure 5, the integration is simpler than in the cloud scenario. In this integration, the loosely coupled architecture provides only additional overhead since the implementation is to be achieved within a single node. A messaging queue system can be omitted in this scenario. Data adapters can connect directly to the data sources (i.e., via HTTP API). Data is transferred between pre-processing, fusion and modeling components directly—via internal interfaces. All the components, including modeling, are implemented using QMiner framework. Incremental learning algorithms, like recursive linear regression and VFDT, are used. Predictions are exposed via lightweight WebSocket protocol and made available in the GUI. In another setup, we have used a lightweight message queue solution based on the MQTT protocol.

## 6. Experimental Results

In this section we present experimental results on a selection of three different use cases from smart cities domains, demonstrating integrations in cloud and edge scenarios. For each use case, we have obtained a real-world dataset, i.e., sensor measurements from the actual testbed. We used non-parametric, linear models (e.g., ridge regression), as well as nonlinear models (e.g., k-nearest neighbours, decision trees, gradient boosting regression and random forests) of different complexities. In the attempt to reach the best possible model performance, we added additional data sources to the model and enriched it with various autoregressive derivatives. These additional data sources are: seasonable date-time related variables (e.g., hour of the day, day of the week), meteorological variables (current and forecasted weather), and additional static variables related to the use case (such as holidays status). To incorporate also different short term trends from the sensor values, we added various autoregressive features, computed for different rolling/sliding windows sizes. Relevant aggregate functions are *mean*, *minimum*, *maximum*, *sum*, and *variance*. Time windows that we used are: *1 h*, *6 h*, *1 day*, *1 week* and *1 month*. Due to the cyclic behaviour, we know that the features from yesterday, or from the same time in the previous week, can be similar to the current values, and are therefore useful for the model. Therefore, some past feature values (*1 day*, *2 days*, and *1 week back*) were also included as autoregressive features.

With respect to the data sources, there are the following universal denominations that we use in the text (for example, data set with name M_AR_WC_WF means that measurements with autoregressive derivatives, current weather and forecasted weather features are included in the dataset):

- **M**—Available sensor measurements (cleaned and resampled)
- **AR**—Autoregressive variables (measurements and their historical and aggregated values)
- **WC**—Current weather
- **WF**—Weather forecasts
- **DT**—Date-time (calendar) properties
- **TOP_20**—20 most important features (obtained with feature selection process)

Evaluation of data fusion algorithms is a topic that has not yet been addressed well even in the traditional batch scenarios. The issue remains an open challenge [1,32]. The data fusion algorithms are usually tested in a simulation environment with unclear performance benefits in a real-world setting. It has been shown that only 1 in almost 20 research papers focuses on the evaluation, relevant to practical applications [60]. To the best of our knowledge, no standardized methodology exists for comparing stream fusion techniques and this remains an open research challenge. Potential evaluation methodology should assess the expressiveness of the feature vector description language (i.e., number of available stream aggregates, ability to utilize historical values, derivatives and aggregates, ability to generate new features by transforming existing ones, ability to include different types of data sources, ability to perform feature selection on-line etc.) as well as how does it benefit the real world applications (i.e., by improving the models, ease of use, number of hyper parameters to be defined, ease of connectivity, initialization time, robustness etc.).

In our evaluation, we show that our methodology has helped to improve modeling capabilities, which is an indirect measure of its benefits in the real world real-time systems. Our system includes six different stream aggregate operators, it can use different historical values and aggregates, it is able to generate new features by applying the difference between historical values and it can integrate three different types of data streams. It can be implemented by a single JSON config file of data sources, feature vectors and model parameters, and it supports connectivity via Apache Kafka, MQTT or REST API. Initialization of the system is dependent on historical data needed for feature vector construction. The system can not estimate historical data based on available data, which in the case of a requirement to include a week old value, will not produce a feature vector until 1 week of viable measurements are in the system.

In Section 6.3 we compare our framework against the ISDI framework [8]. Other methodologies that use values and derivatives (i.e., Kalman filter based methods or other machine learning approaches) can (in the best case) achieve the performance of autoregressive features (denoted with M_AR in the subsections below).

### 6.1. Cloud Infrastructure Deployment for Smart Grid

In this section, we present results from two separate use cases related to electricity distribution. The first case shows the application of our methodology in the smart grid, the second case shows the modeling results from power stations supporting public trains.

**Figure 6:**
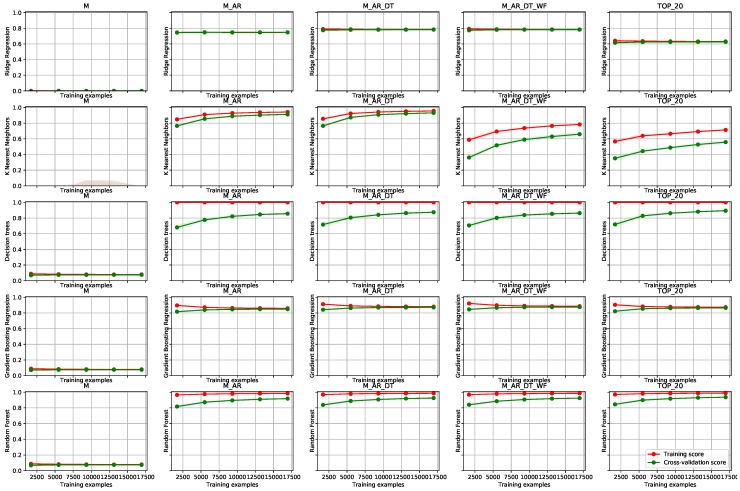
Learning curves on the smart grid use case. Columns depict five different feature vector definitions: M—measurements, M_AR—measurements and autoregressive features, M_AR_DT— measurements, autoregressive features and data-time features, M_AR_DT_WF—all of the features from M_AR_DT and weather forecasts, TOP_20—best 20 features. The rows include results from different learning algorithms: ridge regression, k-nearest neighbours, decision trees, gradient boosting and random forest, respectively. Each sub-figure in the matrix presents a number of training examples on *x* axis and $R^{2}$ score in the *y* axis. The green line represents the learning curve on the test data. The red line represents the learning curve on the train data. The darker band around the curves depicts standard deviations of the $R^{2}$ score.

The **smart grid** use case includes results on predicting measured power from smart meters at five industrial consumer sites. Mean load at each smart meter was 10kW. Testing data set included two full years of data with hourly resolution. We have tested the short-term load forecast scenario (as defined by the energy domain) with a prediction horizon of 10 h. Results are depicted in Figure 6.

The learning curves compare the performance of a model on training and test data over a varying number of training instances. In Figure 6, the red learning curves represent the training score and the great learning curves represent the test score in terms of $R^{2}$. Training score is calculated on a training data set and test score (or cross-validation score) is calculated on a testing set by using cross-validation. In the experiments, we use cross-validation with 10 iterations to get smoother mean test and train score curves, each time with 20% data randomly selected as a testing set (i.e., 80:20 train-test ratio). The shaded area around each curve represents the standard deviation from mean test scores of each step in the cross-validation.

Learning curves offer a better overview of the trained models and allow a data analyst to diagnose, whether the model is trained well or if it has some weakness. The latter can be improved by optimizing method parameters, including more data or reducing the number of features. Such adjustments can prevent overfitting or help models achieve optimal accuracy with the given data set. A tight fit between training and cross-validation scores can indicate that the model suffers from high bias (is under-fitted). Horizontal curve and a consistent gap between the scores indicate that a model has learned as much as it can about the data (additional data would not help). We can see that this is the case for ridge regression and gradient boosting regression. In such cases, one of the standard ways to improve the performance of a model that is suffering from a high bias, is by adding additional informative features or by optimising model parameters. Indeed we can observe that the score has increased using more features (comparison between feature sets from first to the fourth column in Figure 6). A wide gap between training and cross-validation test scores usually indicates that the model is dealing with high variance (over-fitting) problem. This is clear for decision trees, which completely reflect the training data (an almost perfect score is depicted by the red curve), but do not generalize well. In such cases, we might improve our model by obtaining more training examples, or by decreasing model complexity (by decreasing number of features, or model parameter optimisation—i.e., by using shallower trees). Ideally, we want to find a sweet spot that minimizes bias and variance, by finding the right number of features and the right level of model complexity. Among all the possibilities we chose random forests model. Its test scores are the highest (for both, training and cross-validation) and they converged to a constant training score, with more or less all data sets. A tight gap between the testing and training set also indicates that the model generalizes well with new data. Regarding the features, we can observe that for most of the algorithms adding auto-regressive features of the input measurements increased the model performance the most. Static date-time features additionally increased the performance for most algorithms, while weather forecast features don’t seem to affect the modeling results. Nevertheless, we have to take into account that these scores are averaged over the entire testing set. Cases where special features such as bad weather or holidays help are rare. The improvement of the test scores is modest in this case, but correct prediction in these rare cases is valuable as it exposes a deviation from the normal behaviour.

**Figure 7:**
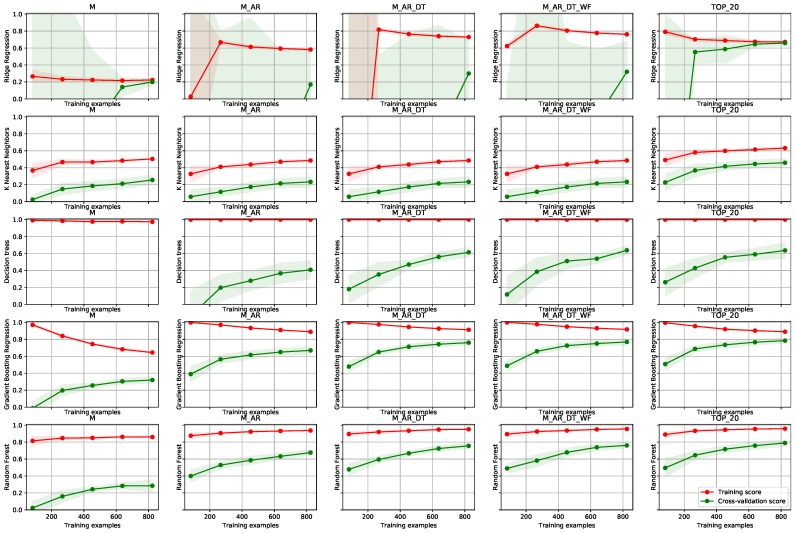
Learning curves on the train substation feeder use case. The structure of the figure mirrors the one from Figure 6.

The second use case presents experiments with train substation feeder with a mean load of approximately 50kW. The data set includes 2 months of measurements with hourly resolution. Again, the prediction horizon has been set at 10 hours. Results are depicted in Figure 7.

Figure 7 clearly depicts that the ridge regression model was the worst model in this use case, with high standard deviation and a large gap between training and cross-validation test scores, which indicates high variance (over-fitting). A high gap between training and cross-validation test scores can be observed in results for decision trees. We can observe that more training examples improve the overall performance of the model, however, the gap does not converge. This indicates that the model can still be improved using more training data. The more or less consistent gap between training and testing scores can be observed with k-nearest neighbors (KNN) and random forest models, but the score is still increasing with more data, which again shows the more data could improve the overall results. But since KNN converged to much lower $R^{2}$ score than random forest, the latter would, of course, be the best choice. Regarding the features, we can observe the same pattern as with previous experimental results in Figure 6. Each additional feature set (data source) slightly improved the performance of the model, which suggests the benefits of our data fusion methodology for the modeling. On the other hand, keeping only the 20 most important features worked very well, since more or less all of the models deal with high variance (poor generalization).

Our framework provides out-of-the-box capabilities for the inclusion of different data sources (see Algorithm 3) and of the corresponding historical (see Algorithm 2), aggregated and aggregated historical values (see Algorithms 1 and 2). Inclusion of new features is possible with a single line in the use-case’s configuration structure. Without extensive additional work, which would implement particular aggregating functions and book-keeping capabilities, in most other systems the modeling results would not exceed the ones, achieved with M or M_AR datasets. ISDI framework, which is the closest to ours in terms of functionality, provides windowing and fusion of multiple data streams, however, extraction of aggregated values is achieved with a custom user-defined function, which is batch-based and not optimized to the incremental nature of the IoT data. Additionally, ISDI’s parformance deteriorates drastically with larger time windows. Our framework already provides built-in mechanisms for the enrichment and generalized inclusion of historical values (without any limitations on the size of windows).

### 6.2. Edge/Fog Infrastructure Deployment on Public Trains

**Figure 8:**
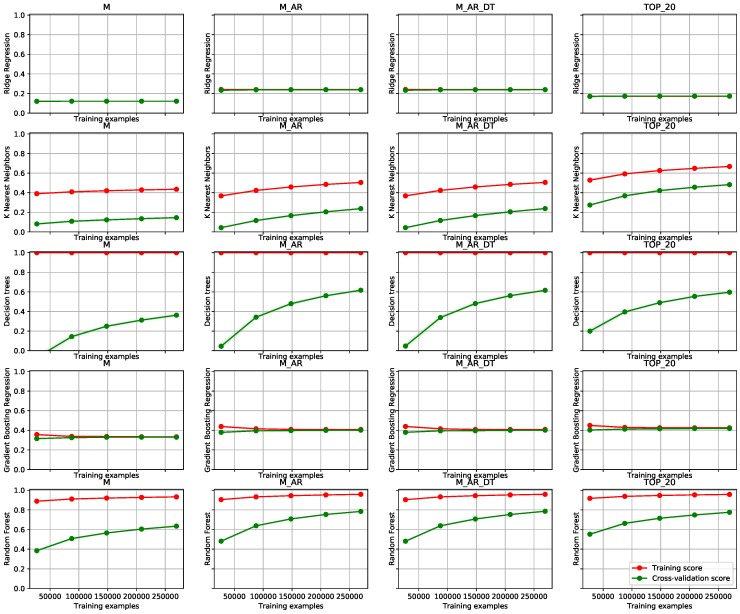
Learning curves on the train autonomous node use case. The structure of the figure mirrors the one from Figure 6, with the exception that weather data set (M_AR_DT_WF) is not included.

The third use case demonstrates the usage of our methodology for stream data fusion, as well as for incremental learning, directly on an edge device where the measurements were taken (we have used Raspberry Pi 3) **on board a public train**. The main reason for this choice was because of the large amount of streaming data at a relatively fast pace. The experiments have been conducted on a data set containing 2 months of data with 1 second time resolution. The task was to predict the power consumption of a train with a very short term prediction horizon (10 s). The results are depicted in Figure 8.

From Figure 8, we can observe that the positive trend of a test set score is rising in every subfigure. It is obvious, that the learning data set has been too small in this case. The benefits of our methodology are, however, apparent and can be observed in a general improvement of the test score with the number of used features (see differences in columns 1 through 3). With the KNN learning method, one can also observe drastic improvement with the data set with selected top 20 features. This exposes the need for a good feature selection methodology, which we have not yet implemented. Decision trees (again) show obvious overfitting to the training data set, however, the improvement of modeling is apparent. In this case, gradient boosting (we used the implementation in scikit-learn) shows poor adaptation to the data and exhibits that it can not be improved with more training data, only with better feature engineering. The method of choice in the experiments is random forest, which achieves the highest test scores with any selected data set.

With the application of Algorithms 1 and 2 we improved the modeling results from the ones depicted in the first (M) to the ones depicted in the second (M_AR) column of Figure 8. With Algorithm 3 we included additional sources and achieved even better modeling results (see column M_AR_DT in Figure 8). With the simple configuration capabilities (https://github.com/klemenkenda/iot-fusion/blob/master/conf/train.js) of the framework, custom feature sets like TOP_20 were implemented and deployed to the production in a matter of minutes.

### 6.3. Performance Tests

**Figure 9:**
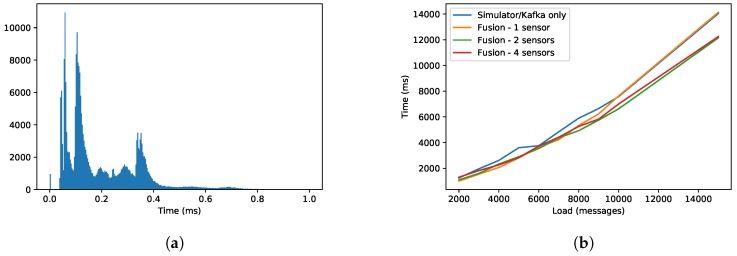
Performance results of data fusion in the smart grid scenario. (**a**) The histogram represents the time used for processing a single message in the data fusion system. A message can be sensor, static or weather forecast data. (**b**) Load graph represents measured processing time of the data fusion system integrated into the Apache Kafka pipeline at different loads and numbers of sensors.

Applicability of the stream fusion framework has been tested from the performance perspective. The results are depicted in Figure 9. The first performance test has been conducted on a real-world smart grid use case data set with $10^{6}$ messages. The setup included streaming fusion of three different types of sources (sensor, static and weather forecasts) and has generated a single feature vector (with 96 features based on current and historical aggregated values on 1-h to 1-month sliding windows) for 24-h prediction horizon. Response times of the fusion component (without modeling) have been measured and the results are depicted in the histogram in Figure 9a. We can observe three major peaks in the histogram. Each of the peaks represents a data source. The peak with the lowest response time corresponds to static data (the simplest data source), the middle one corresponds to weather data (long message with more complex integration subtasks) and the last peak corresponds to sensor data, which in some cases trigger the most computationally demanding fusion process. Median response time of the fusion component is approximately 0.21 ms, which means that the system is able to process approximately 5000 messages per second with a single thread process on an older high-end server (Intel Xeon CPU E5-2667 v2—3.3 GHz, 128 GB RAM, Windows Server 2012 R2). Each process could support the same performance. The measured throughput of Raspberry Pi 3 was approximately eight times slower (700–800 messages per second).

A typical frequency in the smart grid scenario is 15 min. This means that data fusion itself could support a smart grid with up to $18\times 10^{6}$ messages per hour, which corresponds to $3\times 10^{6}$ smart grid nodes per logical processor. ISDI [8] reports (in the best possible scenario) on the throughput of approx. $27\times 10^{6}$ messages per hour per logical processor. The reported throughput is 50% faster than of our framework but it is computed for a single fixed time window (and the feature extraction function is unknown in their evaluation). Also, ISDI encounters major throughput breakdown with larger time windows (>10 days), which is not the case for our methodology. On the other hand, our methodology computes multiple window sizes at the same time (from 1 h up to 1 month) as well as uses multiple aggregate functions. Window sizes can easily be expanded to 1 year with a very little performance cost.

The obvious bottleneck in such a setting is, however, not the fusion algorithm but the ML prediction algorithm. Using the usual method of choice in energy/environment related scenarios (RandomForest with 10 estimators) on the high-end server resulted in one prediction generated per approx. 0.5 s, which roughly corresponds to 7000 smart grid nodes. According to presented integration architectures, the capabilities can be scaled horizontally (over server cores and over other servers) and the final throughput is limited by the state-of-the-art message distribution frameworks such as Apache Kafka, which is apparent in Figure 9b.

## 7. Conclusions and Future Work

The paper describes a novel generic framework for building feature vectors for machine learning from heterogeneous streaming data sources on-line. The main benefit of this methodology is its universal applicability in real-world use cases. It also enables easy configuration of streaming data fusion and modeling pipelines as well as horizontal scalability due to the design patterns used in the architecture.

We have developed and used this methodology in a plethora of use cases, related mostly to efficient energy use, where regressive predictive models have been implemented at the end of the analytical pipeline. In the paper, we have demonstrated the usability of the methodology with three applications in use cases related to smart grids and transport, which are implemented in the cloud and in the edge computing device. The experimental results show that the proposed methodology improves modeling capabilities of the real-world IoT systems.

Future work in this domain should be dedicated firstly to a definition of a thorough evaluation methodology for streaming data fusion frameworks. In this paper, we have relied on an indirect approach with modeling. However, as discussed at the beginning of Section 6, a more thorough approach should be developed, which would take different aspects of data fusion into account.

We see several lines of possible extensions of the proposed framework as follows. Further simplification of data streams might contribute to the easier implementation and faster computation of results (i.e., static data, which is currently included as a stream, might be encoded with a function). Feature selection, dimensionality reduction, instance selection, instance reduction and concept drift are important research topics in the field and should be addressed in the framework. Some of the presented algorithms offer further optimization in terms of performance and expressiveness of the language for describing feature vectors. Stream discretization techniques (implemented via stream aggregates) could be further expanded to match the richness of their batched counterparts. Deployment into a large scale real-world use case states additional challenges related to the management of a large number of data fusion components and models, communications, etc. An efficient deployment system is crucial to ensure the practical scalability of the methodology. In many heterogeneous environments access control is of the utmost importance. Integration of the framework with state-of-the-art methods could be beneficial in various use cases (i.e., healthcare). Finally, the system’s usage depends on the implemented incremental learning algorithms. As mentioned in the introduction, the implementations of the state-of-the-art non-linear incremental learning algorithms are scarce.

Taking into account the shortcomings mentioned above, the framework should find its place in the real-world applications within the IoT and bridge the current gap between academic achievements and practice.

## Abbreviations

The following abbreviations are used in this manuscript:

API	Application Programming Interface
DB	Database
EMA	Exponential Moving Average
GUI	Graphical User Interface
IoT	Internet of Things
ISDI	IoT Streaming Data Integration
JSON	JavaScript Object Notation
KNN	K-Nearest Neighbors (algorithm)
MA	Moving Average
MQTT	Message Queuing Telemetry Transport
REST	Representational State Transfer
RF	Random Forest
SPE	Stream Processing Engine
VFDT	Very Fast Decision Trees (also Hoeffding trees)
VHT	Vertical Hoeffding Trees
